# Immunisation with ‘naïve’ syngeneic dendritic cells protects mice from tumour challenge

**DOI:** 10.1038/sj.bjc.6604221

**Published:** 2008-02-05

**Authors:** M J Grimshaw, K Papazisis, G Picco, H Bohnenkamp, T Noll, J Taylor-Papadimitriou, J Burchell

**Affiliations:** 1 Breast Cancer Biology Group, King's College London School of Medicine, Guy's Hospital, London SE1 9RT, UK; 2 Faculty of Technology, Universitaet Bielefeld, Universitaetsstrasse 25, Bielefeld 33501, Germany

**Keywords:** dendritic cell, immunotherapy, breast cancer, MUC1

## Abstract

Dendritic cells (DCs) ‘pulsed’ with an appropriate antigen may elicit an antitumour immune response in mouse models. However, while attempting to develop a DC immunotherapy protocol for the treatment of breast cancer based on the tumour-associated MUC1 glycoforms, we found that unpulsed DCs can affect tumour growth. Protection from RMA-MUC1 tumour challenge was achieved in C57Bl/6 MUC1 transgenic mice by immunising with syngeneic DCs pulsed with a MUC1 peptide. However, unpulsed DCs gave a similar level of protection, making it impossible to evaluate the effect of immunisation of mice with DCs pulsed with the specific peptide. Balb/C mice could also be protected from tumour challenge by immunisation with unpulsed DCs prior to challenge with murine mammary tumour cells (410.4) or these cells transfected with MUC1 (E3). Protection was achieved with as few as three injections of 50 000 naïve DCs per mouse per week, was not dependent on injection route, and was not specific to cell lines expressing human MUC1. However, the use of Rag2-knockout mice demonstrated that the adaptive immune response was required for tumour rejection. Injection of unpulsed DCs into mice bearing the E3 tumour slowed tumour growth. *In vitro*, production of IFN-*γ* and IL-4 was increased in splenic cells isolated from mice immunised with DCs. Depleting CD4 T cells *in vitro* partially decreased cytokine production by splenocytes, but CD8 depletion had no effect. This paper shows that naïve syngeneic DCs may induce an antitumour immune response and has implications for DC immunotherapy preclinical and clinical trials.

The growth and metastatic spread of tumours depends in part on their ability to evade host immune surveillance and overcome host defences. All tumours express antigens that can be recognised by the immune system, but in most cases an inadequate immune response is elicited because of ineffective activation of effector cells by downregulation of major histocompatibility (MHC) molecules or inhibition of their function by factors produced by the tumour, such as TGF-*β* or IL-10 (Cheng *et al*, 2004; Houghton and Guevara-Patino, 2004; Campoli *et al*, 2005).

Immunotherapy using autologous dendritic cells (DCs) pulsed with tumour-specific antigens has been proposed as a therapeutic strategy for the treatment of a wide variety of tumours, and many preclinical and clinical trials of this approach using different antigens – including peptides and RNA – are underway (Meidenbauer *et al*, 2001; Schuler *et al*, 2003; Wierecky *et al*, 2006). Dendritic cells are potent antigen-presenting cells (APCs), which initiate and regulate innate and antigen-specific responses (Reis e Sousa, 2006). They originate from the bone marrow and their precursors home in via the bloodstream on almost all organs, where they are found in an immature state with high endocytic and phagocytic capability, continuously sampling the antigenic environment. Upon contact with an antigen, such as bacterial DNA, viral dsRNA, microbial products or with inflammatory cytokines, these interstitial DCs change their phenotype and function and migrate to the germinal centres of regional lymph nodes, where they present antigens to the resting or naïve T cells and induce antigen-specific T-cell responses.

MUC1, a highly glycosylated transmembrane glycoprotein, is overexpressed and aberrantly glycosylated by breast cancer cells resulting in changes in the antigenic profile of the tumour-associated glycoforms. In addition, T cells reactive with an HLA-A2^*^0201 class I epitope overlapping the MUC1 signal sequence (M1.2 epitope) have been demonstrated in breast cancer patients with this HLA type (Brossart *et al*, 1999; Beckhove *et al*, 2004; Correa *et al*, 2005). Mouse MHC class I epitopes particularly for H2^b^, as well as for other allotypes, have also been shown to be present in MUC1 (Apostolopoulos *et al*, 1998; Heukamp *et al*, 2001). Furthermore, it has been shown that lysate-pulsed DCs can cross-present the M1.2 epitope effectively and cross-prime CD8+ T cells *in vitro* (Bohnenkamp *et al*, 2004). Although it is not completely clear which components of the innate or adaptive immune system can be mobilised by different MUC1 immunogens and which are crucial for tumour rejection, evidence is accumulating that inhibition of tumour growth in mice and humans can be achieved. There are several reports presenting data from mouse models that suggest that loading DCs with MUC1-based immunogens can result in tumour rejection in MUC1 transgenic (MUC1.Tg) mice (Koido *et al*, 2000; Chen *et al*, 2003).

It has previously been reported that loading murine bone marrow-derived DCs with a MUC1 peptide and injecting them into mice protects the animals from subsequent challenge with MUC1-expressing breast tumours (Soares *et al*, 2001). In this paper, we present data that were obtained while attempting to pursue these observations and which show that protection from tumour challenge can be obtained using DCs that have not been loaded with any tumour-specific antigen. Using a different model system, a similar observation has been reported by the group of Berzofsky (van den Broeke *et al*, 2003). This phenomenon has important implications for the design of both pre-clinical and clinical DC-based protocols.

## MATERIALS AND METHODS

### Cell lines

Murine 410.4 (murine epithelial breast tumour cells derived from Balb/C mice) cells were a gift from Bonnie Miller (Michigan Cancer Foundation, Detroit, MI, USA) and were cultured in DMEM medium supplemented with 10% FCS and 25 *μ*g ml^−1^ insulin. E3 cells, which express human MUC1 from its own promoter, were generated from 410.4 cells as described by Lalani *et al* (1991). RMA (murine leukaemia virus-induced T-cell lymphoma) cells were transfected with human MUC1 under the control of a cytomegalovirus (CMV) promoter and cultured as described previously (Plunkett *et al*, 2004).

### Mouse strains

Female Balb/C and C57Bl/6 mice and transgenic mice (approximately 10 weeks of age) were used for bone marrow harvest and tumour challenge. MUC1.Tg mice expressing human MUC1 from the human MUC1 promoter, homozygous for the transgene expression, were originally developed on an H2-k background (Peat *et al*, 1992). These mice have been backcrossed onto C57Bl/6 and Balb/C strains for 15 generations to give a pure C57Bl/6 (H2^b^) or pure Balb/C (H2^d^) backgrounds. Recombination activation gene 2 (Rag2)–knockout (Rag2^−/−^) mice are on a C57Bl/6 background.

### Generation of DCs

Bone marrow was isolated from the femurs and tibias of 8–12 weeks old female mice. Cells were resuspended in AIM-V medium in plastic culture dishes for 45 min; adherent cells were then discarded. Non-adherent and loosely adherent cells were resuspended in AIM-V medium supplemented with 50 *μ*M *β*-mercaptoethanol and 10 ng ml^−1^ IL-4 and GM-CSF (R&D Systems, Abingdon, UK). Fresh cytokines were added after 2 days culture. After 3 days of culture, non-adherent cells were removed and fresh medium supplemented with cytokines was added to the adherent cells. After a further 4 days, during which fresh cytokines were added every 48 h, non-adherent cells were collected and, where appropriate, primed with peptide. Dendritic cells were washed three times in endotoxin-free PBS prior to injections.

### Flow cytometry

FITC- and PE-labelled mAbs against DC markers (BD Biosciences Pharmingen, San Jose, CA, USA) and isotype-matched labelled controls were used to characterise cell surface phenotypes by flow cytometry.

For staining, cells were washed and resuspended in PBS supplemented with 1% heat-inactivated FBS and 0.01% NaN_3_. Antibodies were diluted in this buffer and used at a final concentration of 25 *μ*g ml^−1^. Incubations with antibodies were carried out for 45 min on ice. Following washing, labelled cells were fixed with 1% formaldehyde solution and 10 000 cells were analysed by flow cytometry.

### Pulsing DCs

DCs were washed and resuspended in fresh medium with cytokines at a density of 10^6^ cells per ml. Dendritic cells were ‘pulsed’ overnight with a 60-mer peptide consisting of three tandem repeats of MUC1 (20 *μ*g ml^−1^). After pulsing, cells were washed three times in PBS.

### Immunisation and tumour challenge

For protection experiments, mice received three subcutaneous injections (a week apart) of PBS containing (unless otherwise indicated) 5 × 10^4^ DCs into the flank. A control group of mice were injected with PBS only. A week after the final DC injection, 0.1 ml PBS containing tumour cells (5 × 10^5^ 410.4 or E3 cells; 5 × 10^4^ RMA-MUC1 cells) was injected subcutaneously into the flank of Balb/C (E3 and 410.4) or C57Bl/6 WT or transgenic mice (RMA-MUC1). For ethical reasons, mice were sacrificed when tumours reached 1.44 cm^2^. UKCCCR guidelines were followed at all times and all animal work was performed under Home Office Project licence No. PPL 70/4701.

### Microchemotaxis assay

Chemotaxis was examined using a 48-well microchemotaxis chamber (Neuro Probe, Cabin John, MD, USA). The lower wells were filled with RPMI supplemented with 250 ng ml^−1^ chemokine and covered with an 8-*μ*M-pore polycarbonate membrane. Cell suspension (100 000 DCs in 100 *μ*l serum-free medium) was added to each upper well. After incubation for 2 h at 37°C for 90 min, the membrane was removed and the cells attached to the upper surface of the membrane were removed by washing with PBS. The membrane was fixed in methanol and stained with Diff-Quik. Cells attached to the lower surface of the membrane or in the lower well of chamber were counted.

### Splenocyte preparation

Spleens were disaggregated and the cells suspended in RPMI medium before centrifugation at 1500 rpm for 10 min. Splenocytes were isolated using a Ficoll–Paque gradient (GE Healthcare UK Ltd, Buckinghamshire, UK), and cell clumps were removed by filtering the cells through 30 *μ*M filters. Cells were washed twice with RPMI and resuspended (10^7^ cells per ml) in RPMI supplemented with 5% FCS and 10 *μ*M *β*-mercaptoethanol, before being plated in a 96-well culture plate (100 *μ*l per well) with or without 10^6^ unpulsed DCs. CD4- or CD8-positive cells were removed *in vitro* prior to culture using commercially available BD IMag Particles (BD Biosciences Pharmingen) according to the manufacturer's instructions.

### Cytokine determination

Cytokine (IFN-*γ* and IL-4) concentrations in cell culture supernatants were measured by ELISAs from R&D Systems according to the manufacturer's instructions.

## RESULTS

### Phenotypic and functional analysis of murine DCs

**Figure 1:**
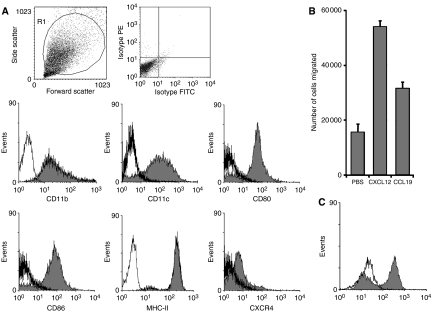
Bone marrow-derived DCs grown in serum-free medium show a typical dendritic phenotype and are functionally active. (**A**) Bone marrow-derived DCs from C57Bl/6 MUC1.Tg mice were cultured for 6 days in serum-free medium and their phenotype determined by flow cytometry. (**B**) Migration towards chemokines of 6-day cultured DCs was measured in the transwell system. (**C**) The ability of DCs to uptake FITC dextran was measured at 4°C (thick line) and at 37°C (shaded).

DCs were cultured from the bone marrow of wild-type or MUC1. Tg mice on Balb/c and C57Bl/6 backgrounds in serum-free medium. The phenotype of the cells was analysed by flow cytometry: cells from all strains of mice were CD11b^+ve^, CD11c^+ve^, CD80^+ve^, CD86^+ve^, MHC class II^+ve^, CXCR4^weakly +ve^ and CD8a^−ve^. Figure 1A shows the phenotype of DCs derived from C57Bl/6 MUC1.Tg mice. Dendritic cells derived from the bone marrow of wild-type C57Bl/6 and Balb/c mice had a similar phenotype (data not shown). The DCs used in this study were therefore phenotypically mature and functionally active as shown by their ability to migrate towards chemokines (Figure 1B) and to take up FITC-dextran (Figure 1C). Pulsing cells with MUC1 peptide did not alter DC phenotype (data not shown). Stimulating cells with either LPS or prostaglandin E2/tumour necrosis factor *α* increased cell surface CXCR4 but did not increase *in vitro* migration (data not shown).

### Vaccination with pulsed or unpulsed DCs can protect mice from tumour challenge

**Figure 2:**
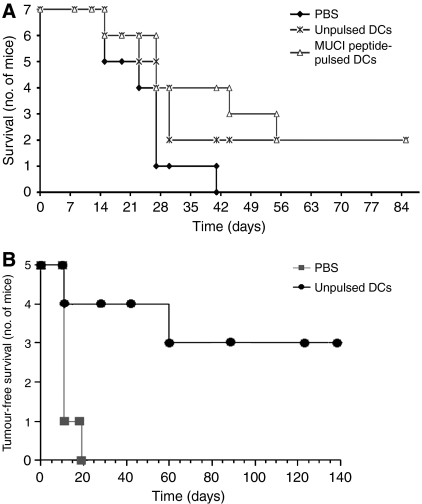
Naïve DCs as well as DCs pulsed with antigen can protect mice from tumour challenge. (**A**) Groups of seven C57Bl/6 MUC1.Tg mice were vaccinated with three injections of syngeneic DCs given 3 weeks apart either pulsed with a 60-mer peptide corresponding to three tandem repeats of MUC1 or naïve unpulsed DCs. Control mice were vaccinated with PBS. At 1 week after the third injection, the mice were challenged with RMA tumour cells expressing human MUC1. For ethical reasons the mice were sacrificed when the tumour reached 1.4 cm^2^. (**B**) Wild-type Balb/C mice (five per group) were given three weekly subcutaneous injections of either PBS or 5 × 10^5^ unpulsed DCs. A week after the final DC injection, mice were challenged with subcutaneous injection of 500 000 E3 breast tumour cells. Shown is one representative experiment of three.

To investigate the ability of DCs pulsed with MUC1 peptides to protect mice from challenge with MUC1-expressing tumours, we vaccinated C57Bl/6 MUC1.Tg. mice with MUC1 peptide-pulsed DCs, similar to the experiments described by Soares *et al* (2001). C57Bl/6 MUC1.Tg mice were vaccinated with three injections, 3 weeks apart, of syngeneic MUC1.Tg DCs, pulsed or unpulsed with MUC1 peptide. One week after the last injection, the mice were challenged with RMA tumour cells expressing human MUC1 under a CMV promoter. Although peptide-pulsed DCs significantly increased the survival of the mice compared with the buffer control group, unpulsed DCs also gave significant protection so that by day 56, the protection from naïve DCs was indistinguishable from that obtained with the pulsed DCs (Figure 2A). This made it impossible to evaluate the effect of immunisation of mice with DCs pulsed with the specific peptide.

To ascertain whether the protection from tumour challenge provided by unpulsed DCs was limited to this model, we changed the mouse strain and the tumour cell line. Wild-type Balb/C mice were treated with three weekly subcutaneous injections of unpulsed wild-type DCs (50 000 cells per mouse per week). A week after the final DC injection, the mice were challenged with E3 tumour cells (the murine mammary tumour cell line 410.4 transfected with human MUC1; Figure 2B). In the PBS control groups, all mice had a tumour within 3 weeks of tumour challenge. However, unpulsed DCs significantly increased the tumour-free survival of the mice, with 60% of the mice remaining tumour-free until the termination of the experiment at 140 days. Thus, in two different murine models, immunisation with naïve DCs can protect mice from tumour challenge.

### Protection is dependent on the number of injected DCs

**Figure 3:**
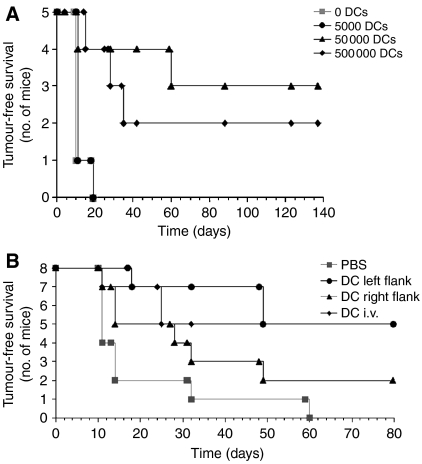
Protection from tumour challenge is dependent on the number of DCs injected but not on the route of injection. (**A**) Balb/C mice were injected three times with 0, 5 × 10^3^, 5 × 10^4^ or 5 × 10^5^ DCs per mouse per week prior to tumour challenge with E3 cells. (**B**) Three weekly injections of 5 × 10^4^ DCs were administered to wild-type Balb/c mice subcutaneously in the right or left flank or intravenously into the tail vein. At 1 week after the final injection, the mice were tumour challenged with E3 cells.

We analysed the effects of different numbers of DCs on tumour protection in two different mouse strains. Wild-type Balb/C mice were vaccinated with zero to 500 000 DCs per week and after three DC injections, mice were challenged with the E3 tumour. Injections of 5000 DCs per mouse per week did not protect the mice from subsequent tumour challenge (Figure 3A). However, injection of 50 000 or 500 000 resulted in a significant number of mice remaining tumour-free up until the termination of the experiment at day 140. Increasing the number of DCs above 50 000 did not further improve tumour protection.

### Protection is not compromised by route of injection

In the previous experiments, immunisation injections were at the same site as tumour challenge (ie, subcutaneous injections in the left flank). We next tested whether the tumour challenge in the same site as DC injection effectively ‘pulsed’ the DCs *in vivo*, or whether an inflammatory effect at the tumour challenge site was ‘activating’ DCs, by varying the route of injection. Balb/C mice were given three injections of unpulsed DCs or PBS into either the right or left flank; DCs were also injected intravenously. The subsequent E3 tumour challenge was injected into the left flank of all groups. Injection of unpulsed DCs gave a similar level of tumour protection whether the injection site was left flank or intravenous (Figure 3B), indicating that tumour protection does not derive from the synergy of the DCs and an inflammatory response or from ‘*in vivo* pulsing’.

### Protection from tumour challenge is not dependent on MUC1 but does require T or B cells

**Figure 4:**
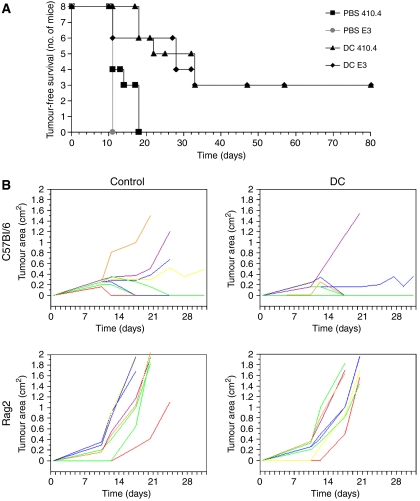
Protection from tumour challenge is not dependent on MUC1 expression but does require B or T cells. (**A**) Wild-type Balb/C mice (eight per group) were given three weekly subcutaneous injections of either PBS or 5 × 10^5^ unpulsed DCs. A week after the final DC injection, mice were challenged with subcutaneous injection of either 410.4 or E3 cells (410.4 transfected with human MUC1) (500 000 tumour cells per mouse). (**B**) C57Bl6 and Rag2^−/−^ mice were vaccinated with 50 000 DCs (three times, 1 week apart) before challenge with the RMA tumour cell line.

The E3 murine mammary tumour cell line expresses human MUC1. To test whether DC-mediated protection from tumour challenge depended on the presence of a foreign antigen in the tumour cells (ie, human MUC1), we tumour challenged naïve DC immunised mice with E3 cells or parental wild-type 410.4 cells. Wild-type Balb/C mice were immunised with unpulsed DCs derived from wild-type Balb/C bone marrow prior to tumour challenge. As previously observed, naïve DCs protected Balb/C mice from E3 tumour challenge. However, mice were also protected from challenge with the syngeneic parental cell line, 410.4 (Figure 4A).

To investigate the mechanism involved in the tumour protection, we immunised Rag2^−/−^ mice with naïve DC and tumour challenged with RMA cells. The phenotype of Rag2^−/−^ mice, which are on a C57Bl6 background, is mature T- and B-cell deficiency. Although the Rag2^−/−^ mice developed tumours more quickly than the wild-type C57Bl6 mice, by day 13 all the Rag2^−/−^mice in the control group and the naïve DC group had developed tumours and there was no difference in the tumour growth between the two groups (Figure 4B). In contrast, in the wild-type mice, vaccination with naïve DCs reduced the take and growth of the RMA tumour cells compare with the wild-type controls (Figure 4B).

These data suggest that either T or B cells are involved in the tumour protection observed with naïve DC cells and that the protection is not dependent on the presence of a foreign antigen.

### Naïve DCs induce the production of IFN-*γ* and IL-4 by splenocytes, which is dependent on T-cell function

**Figure 5:**
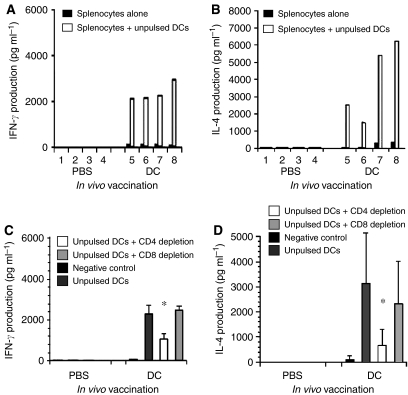
Unpulsed DCs can stimulate *in vivo* primed splenocytes to secrete cytokines *in vitro*. Mice were injected three times with either PBS or naïve DCs; 1 week after the last injection, the splenocytes were isolated and cultured for 48 h in the presence or absence of naïve DCs and the cytokines produced measured by ELISA. (**A**) IFN-*γ* production by splenocytes from mice immunised with DCs or PBSA cultured *in vitro* in the presence (clear bars) or absence (black bars) of naïve DCs. Measurements from eight individual splenocyte preparations are shown; (**B**) IL-4 production by splenocytes from mice immunised with DCs or PBSA cultured *in vitro* in the presence (clear bars) or absence (black bars) of naïve DCs. Measurements from eight individual splenocyte preparations are shown. (**C** and **D**) CD4+ cells or CD8+ cells were depleted from splenocytes of mice vaccinated with naïve DCs prior to *in vitro* culture in the presence or absence (negative control) of naïve DCs. (**C**) IFN*γ* secretion (^*^, *P*<0.05), the mean of four splenocyte preparations±s.d. is shown. (**D**) IL-4 secretion (^*^, *P*<0.05), the mean of four splenocyte preparations±s.d. is shown.

To investigate the immune response induced by naïve DCs, wild-type C57Bl/6 mice were immunised three times with naïve DCs; 1 week after the final injection, the spleens were removed and the splenocytes cultured for 48 h in the presence or absence of syngeneic DCs. Incubation of naïve DCs with splenocytes from mice vaccinated with unpulsed DCs resulted in the secretion of high levels of IFN-*γ* and IL-4 (Figure 5A and B). No IFN-*γ* or IL-4 was detected when DCs were incubated with splenocytes from mice injected with PBS. Thus the vaccination of mice with DCs cultured for 7 days *in vitro*, without exposure to specific antigen, was sufficient to induce the *in vitro* secretion of IFN-*γ* and IL-4. It should be noted that the DCs used to immunise the mice were cultured in serum-free medium, thus eliminating any artefacts due to the foetal calf sera.

To determine the source of the cell producing the IFN-*γ* and IL-4, the CD4+ or CD8+ T cells were depleted prior to the incubation with DCs. The efficacy of depletion was analysed by flow cytometry and shown to be 93.0±0.5% for CD4^+ve^ and 68.4±10.4% for CD8^+ve^ T cells (data not shown). Depleting the splenocytes of CD4 cells partially but significantly decreased the induction of IFN-*γ* (Figure 5C) and IL-4 (Figure 5D) release, whereas depleting CD8 cells had no effect.

### Therapy of E3 tumour with unpulsed DCs

Although protection from tumour challenge has been attained using immunotherapy and vaccination methods, therapy of established tumours is usually less effective. Therefore, having shown induction of protection against the E3 tumour using unpulsed DCs, we tested whether naïve DCs had any effect on the more stringent system of the established tumour. Balb/C mice were challenged with E3 tumour cells and then given three weekly subcutaneous injections of naïve DCs or PBS.

**Figure 6:**
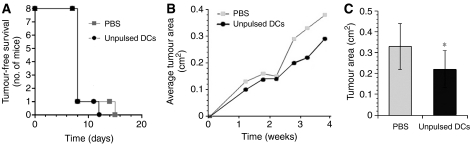
Therapy of E3 tumour-bearing mice by immunisation with naïve DC. Groups of eight Balb/C mice were subcutaneously injected with E3 cells. After 7 days, weekly subcutaneous injections of PBS, unpulsed DCs (10^5^ cells per mouse per week) began, for 3 weeks. (**A**) Tumour-free survival of the mice, (**B**) growth of tumours over time. (**C**) Comparison of the average size of the tumours at day 21. The tumour area was significantly (^*^, *P*<0.05) smaller in the DC-treated group compared with PBS-treated mice.

Immunisation with DCs did not prevent tumour growth (Figure 6A), but tumour growth was significantly less in mice immunised with naïve DCs (Figure 6B and C).

## DISCUSSION

Immunotherapy using autologous DCs pulsed with a tumour-specific antigen has been proposed as a therapeutic strategy for the treatment of a wide variety of tumours, and many preclinical and clinical trials of this approach are underway (Meidenbauer *et al*, 2001). Indeed, several studies have shown an encouraging clinical response in both clinical trials and numerous animal models (Schuler *et al*, 2003; Wierecky *et al*, 2006). One of the critical factors shown to influence the efficacy of DC immunotherapy has been the preparation and differentiation of DCs (Josien *et al*, 2000; Liu *et al*, 2002; Schuler *et al*, 2003). In this study, we have cultured murine bone marrow cells in the presence of IL-4 and GM-CSF to yield high-quality CD11b^+ve^ CD11c^+ve^ CD8^−ve^ myeloid mature DCs that express high levels of stimulatory MHC class II molecules, as well as CD80, CD86 and the chemokine receptor CXCR4. Furthermore, it was demonstrated that the generated DCs are functionally active by migrating effectively towards CCL19 and CXCL12, the ligands for CCL7 and CXCR4, respectively. We show that injection of mice with DCs pulsed with a MUC1 tandem repeat peptide (60-mer corresponding to three tandem repeats) can protect wild-type and MUC1.Tg mice from subsequent challenge with a MUC1-expressing tumour. However, we also demonstrated that injection of unpulsed ‘naïve’ DCs are equally efficient at protecting mice from tumour challenge. We have shown that the protection gained by injection of naïve DCs in our studies is not due to (1) the expression of a foreign (human) antigen, (2) a high number of DCs being injected, (3) the route of immunisation, suggesting the induction of systemic protection, (4) strain of mouse, or (5) tumour model. Using Rag2^−/−^ mice we have demonstrated that the protection observed with naïve DCs was dependent on elements of the adaptive immune response, that is T or B cells.

Moreover, *in vivo* priming with syngeneic unpulsed DCs resulted in the secretion of large amounts of IFN-*γ* and IL-4 upon a single *in vitro* stimulation with naïve DCs.

Interestingly, it has been shown that DCs isolated from mouse spleens spontaneously produce IL-12 and TNF-*α*, upregulate costimulatory molecules and induce the activation of antigen-specific IFN-*γ*-producing CD4+ T cells *in vivo* (Schlecht *et al*, 2006). The responses were similar to those induced by DCs activated by CpG, a strong Th1-promoting adjuvant (Klinman, 2006). Moreover, DCs derived *in vitro* from bone marrow have been shown to secrete type I IFNs that act in an autocrine manner to activate the DCs enabling them to activate T cells (Montoya *et al*, 2002). The DCs used in this study express relatively high levels of CD80, CD86 and class II and possibly were more activated than those used in other studies. Dendritic cells can induce tolerance or immunity according to their activation state (Moser, 2003; Reis e Sousa, 2006); it is possible that DCs activated *in vitro* by the culture conditions could present self-antigens – common to the tumour cells – to T cells resulting in their activation. Certainly, effector T cells have been shown to respond to a lower ligand affinity threshold than naïve T cells, and effector T cells have been shown to respond to endogenous self-peptide presented by APCs (Kimachi *et al*, 2003). Further experiments will be needed to investigate whether there is evidence of induced autoimmunity in our models.

To minimise the exposure of the DCs to foreign proteins, the DCs were cultured in serum-free AIM V medium. However, it cannot be ruled out that the DCs presented proteins found within the medium to T cells *in vivo* and that the *in vitro* cytokine production observed with unpulsed DCs resulted from a stimulation of these cells by the DCs. This could be possible for although the splenocytes were incubated with DCs in a completely different medium (RPMI 10% FCS), the DCs used for the *in vitro* assay were derived from bone marrow in AIM-V medium. However, this cannot explain the ability of naïve DCs to protect mice from tumour challenge, and other groups have used AIM-V to generate bone marrow-derived DCs (Soares *et al*, 2001).

There are many reports in the literature of the use of primed DCs to induce tumour protection in murine models. Most of these (Zitvogel *et al*, 1996; Ashley *et al*, 1997; Boczkowski *et al*, 2000; Irvine *et al*, 2000; Nair *et al*, 2000; Shimizu *et al*, 2001; Koido *et al*, 2002; Chen *et al*, 2003) have included control DC groups, whereas others have not (Soares *et al*, 2001). In reports that included a control group of unpulsed DCs, no significant protection was seen with these cells. However, in agreement with our data, the study by van den Broeke *et al* (2003) showed that unpulsed DCs induced protection against tumour lung metastases. As we have found, this protection was independent of the strain of mouse, tumour source or route of injection of the DCs. Moreover, they showed that CD4+ T cells were necessary for protection together with NK cells. The authors conclude that the DC-mediated NK cell activation was likely to be through an intermediate interaction of DCs with CD4+ T cells rather than a direction effect on the NK cells. Furthermore, although small in number there have been other reports that unpulsed DCs can induce tumour protection (Yang *et al*, 1997; DeMatos *et al*, 1998). Interestingly, early studies by Knight (Knight *et al*, 1985) also suggested that normal syngeneic DCs could induce tumour regression or delayed tumour growth.

From the results presented here it is clear that manipulation of DCs *in vitro* can result in their ability to stimulate an immune response without actively pulsing the DCs with antigen. One of the hurdles in cancer immunotherapy has been to show a connection between any clinical benefit and the antigen with which the DCs were pulsed. In a stage IV melanoma clinical phase I study using four melanoma antigens with BM-derived DCs, none of the patients analysed showed an expansion of melanoma-peptide-specific circulating effector memory T cells, and there were no objective clinical responses (Banchereau *et al*, 2005). However, in other studies, there were detectable levels of CD4-specific Th1 cells and specific CD8 T cells in most but not all of the patients (Schultz *et al*, 2004; Fay *et al*, 2006).

The fact that unpulsed DCs can induce T cells to produce large amounts of cytokines (IFN-*γ* and IL-4) and protect mice from tumour challenge has implications for the use of DCs in immunotherapy. Understandably, unpulsed DCs have not been included in the early clinical trials investigating the efficacy of DCs for the treatment of cancer patients. It is possible that some of the promising results reported are not dependent on the antigen used to pulse the DC *ex vivo*. More importantly, if injection of naïve ‘culture-activated’ DCs could induce T cells to self-antigens, there is a possibility of inducing autoimmunity in patients undergoing such therapy and therefore further research into the results reported here is required.
